# Exercise modulates sympathetic and vascular function in chronic kidney disease

**DOI:** 10.1172/jci.insight.164221

**Published:** 2023-02-22

**Authors:** Jinhee Jeong, Justin D. Sprick, Dana R. DaCosta, Kevin Mammino, Joe R. Nocera, Jeanie Park

**Affiliations:** 1 Division of Renal Medicine, Department of Medicine, Emory University School of Medicine, Atlanta, Georgia, USA.; 2 Department of Veterans Affairs (VA) Health Care System, Decatur, Georgia, USA.; 3 Department of Kinesiology, Health Promotion and Recreation, University of North Texas, Denton, Texas, USA.; 4 Center for Visual and Neurocognitive Rehabilitation, Department of VA Health Care System, Decatur, Georgia, USA.; 5 Departments of Neurology and Rehabilitative Medicine, Emory University Department of Medicine, Atlanta, Georgia, USA.

**Keywords:** Clinical Trials, Neuroscience, Chronic kidney disease

## Abstract

**BACKGROUND:**

Chronic kidney disease (CKD) is characterized by chronic overactivation of the sympathetic nervous system (SNS), which increases the risk of cardiovascular (CV) disease and mortality. SNS overactivity increases CV risk by multiple mechanisms, including vascular stiffness. We tested the hypothesis that aerobic exercise training would reduce resting SNS activity and vascular stiffness in patients with CKD.

**METHODS:**

In this randomized controlled trial, sedentary older adults with CKD underwent 12 weeks of exercise (cycling, *n* = 32) or stretching (an active control group, *n* = 26). Exercise and stretching interventions were performed 20–45 minutes/session at 3 days/week and were matched for duration. Primary endpoints include resting muscle sympathetic nerve activity (MSNA) via microneurography, arterial stiffness by central pulse wave velocity (PWV), and aortic wave reflection by augmentation index (AIx).

**RESULTS:**

There was a significant group *×* time interaction in MSNA and AIx with no change in the exercise group but with an increase in the stretching group after 12 weeks. The magnitude of change in MSNA was inversely associated with baseline MSNA in the exercise group. There was no change in PWV in either group over the study period.

**CONCLUSION:**

Our data demonstrate that 12 weeks of cycling exercise has beneficial neurovascular effects in patients with CKD. Specifically, exercise training safely and effectively ameliorated the increase in MSNA and AIx observed over time in the control group. This sympathoinhibitory effect of exercise training showed greater magnitude in patients with CKD with higher resting MSNA.

**TRIAL REGISTRATION:**

ClinicalTrials.gov, NCT02947750.

**FUNDING:**

NIH R01HL135183; NIH R61AT10457; NIH NCATS KL2TR002381; and NIH T32 DK00756; NIH F32HL147547; and VA Merit I01CX001065.

## Graphical abstract

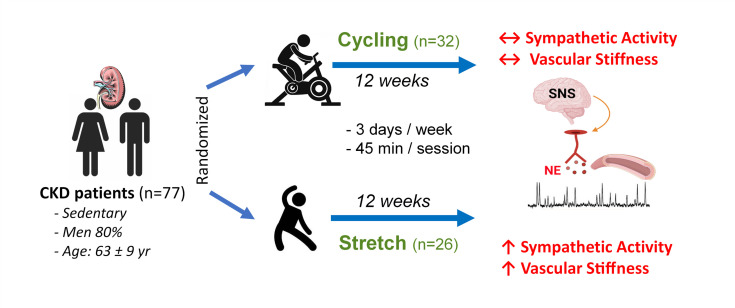

## Introduction

Patients with chronic kidney disease (CKD) are at profoundly increased risk for cardiovascular (CV) disease and mortality (1). Chronic overactivation of the sympathetic nervous system (SNS) is recognized as an important contributor to increased CV risk in patients with kidney dysfunction (2). SNS overactivity presents early in the course of CKD (3–7) and worsens with progression to end-stage kidney disease (8). SNS overactivity independently increases CV risk not only by increasing blood pressure (BP), but also by BP-independent effects on end organs and tissues, including the heart, kidney, muscle, and vasculature (9, 10). Notably, SNS overactivity promotes vascular structural abnormalities and key functional consequences, including arterial stiffening and augmented aortic wave reflection (11, 12). These vascular abnormalities, highly prevalent in CKD, are strong predictors of adverse CV events and mortality (13, 14). Current therapies targeting SNS overactivity are limited to pharmacologic agents such as peripheral and central sympatholytics (i.e., clonidine) that are often poorly tolerated. Thus, therapeutic strategies aimed at reducing SNS overactivity in patients with CKD represent an important clinical goal, aimed at improving autonomic and vascular function to reduce CV risk in CKD.

Exercise training has been shown to decrease muscle sympathetic nerve activity (MSNA) in other clinical populations characterized by elevated SNS activity (15–25). Specifically, patients with heart failure (15–19), metabolic syndrome (20, 21), obesity (22, 23), and hypertension (24, 25) who were engaged in prolonged aerobic exercise training exhibited exercise-induced reductions in resting MSNA. However, the sympathomodulatory effect of exercise training has not been tested in patients with CKD. Furthermore, evidence of exercise-induced vascular adaptation in CKD is conflicting, with some studies showing no improvement (26–28) and others showing improvement limited to the microvasculature (29, 30).

Therefore, we tested the hypothesis that 12 weeks of aerobic exercise training reduces resting SNS activity and improves indices of arterial stiffness (i.e., carotid-femoral pulse wave velocity [PWV] and augmentation index [AIx]) in patients with CKD stages III–IV.

## Results

### Participants.

**Figure 1:**
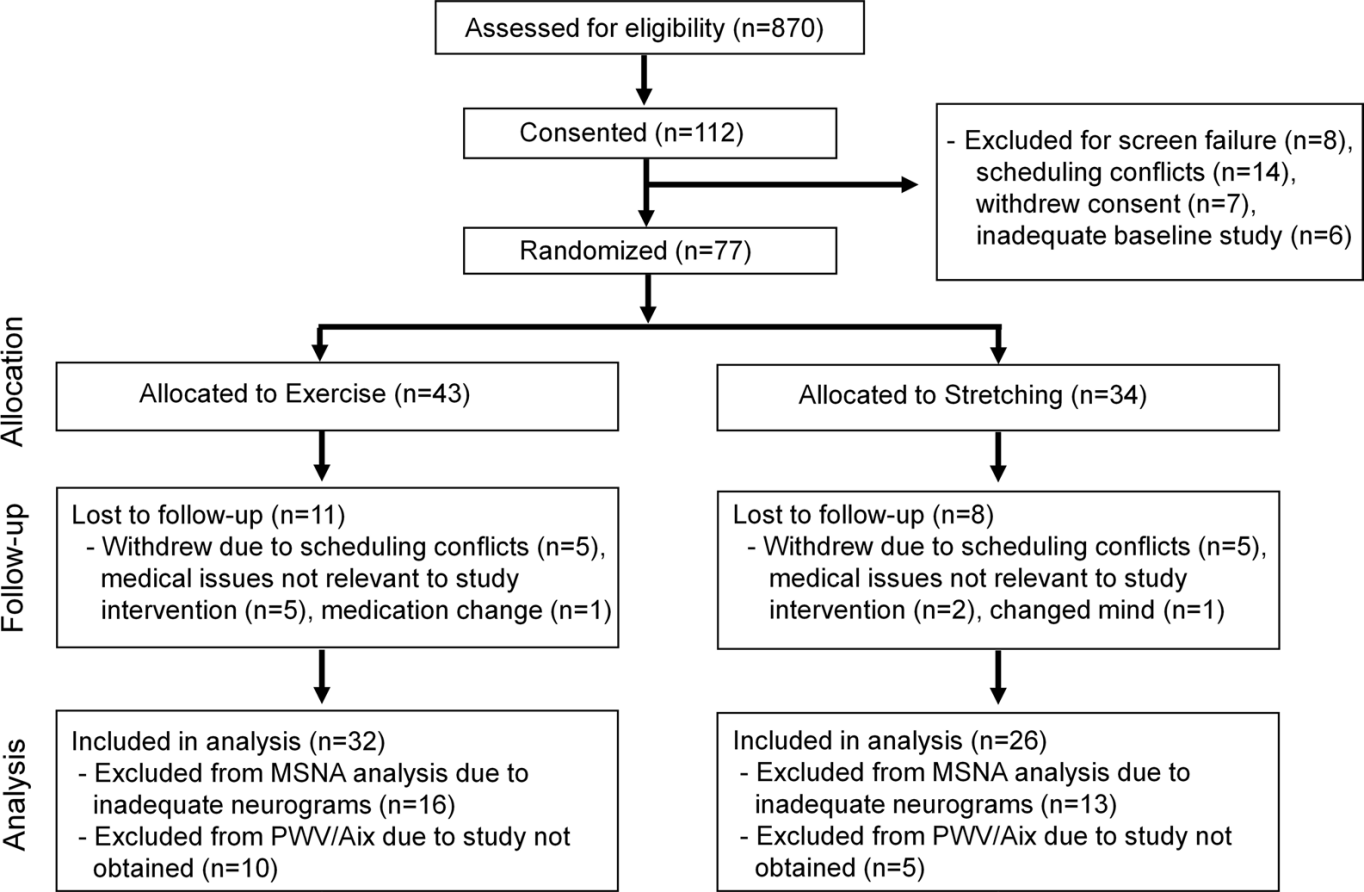
CONSORT diagram. The diagram depicts patient flow through the randomized clinical trial.

A total of 58 participants who completed the study intervention were included in this study, as described in the consort diagram in Figure 1. Participant baseline characteristics are presented in Table 1. The majority of participants in each group were older Black males and had hypertension. All female participants were postmenopausal with a mean and a minimum age of 63.5 ± 6.4 years and 54 years, respectively. There were no differences in demographics, anthropometric characteristics, volume status, medication use, comorbidities, blood chemistry, kidney function, or peak aerobic capacity parameters between groups.

### Intervention adherence, body composition, and exercise capacity.

Intervention adherence measured by attended prescribed sessions and total time spent for the sessions was high in both groups and comparable (Table 1). In total, 90% of participants in the exercise group (Exercise) and 87% of participants in the stretching group (Stretching) completed more than 90% of training sessions (>33 sessions). Overall, 30% of participants in Exercise and 39% of participants in Stretching completed all assigned 36 training sessions. The primary reason for the missed sessions in both groups was a scheduling conflict for the participant. One participant in Exercise completed 2 extra sessions (a total of 38 sessions) to minimize the time gap between the last exercise training session and the testing visits.

There were no significant changes in BMI, body fat percent, or aerobic exercise capacity (VO_2peak_) after 12 weeks in both Exercise (32.6 ± 6.2 kg/m^2^, 34.3% ± 9.2%, and 26.0 ± 6.3 mL/kg/min, respectively; *P* > 0.05 for all) and Stretching (31.9 ± 5.4 kg/m^2^, 31.9% ± 10.3%, and 25.8 ± 6.7 mL/kg/min, respectively; *P* > 0.05 for all).

### Effects of exercise training on resting sympathetic nerve activity.

**Figure 2:**
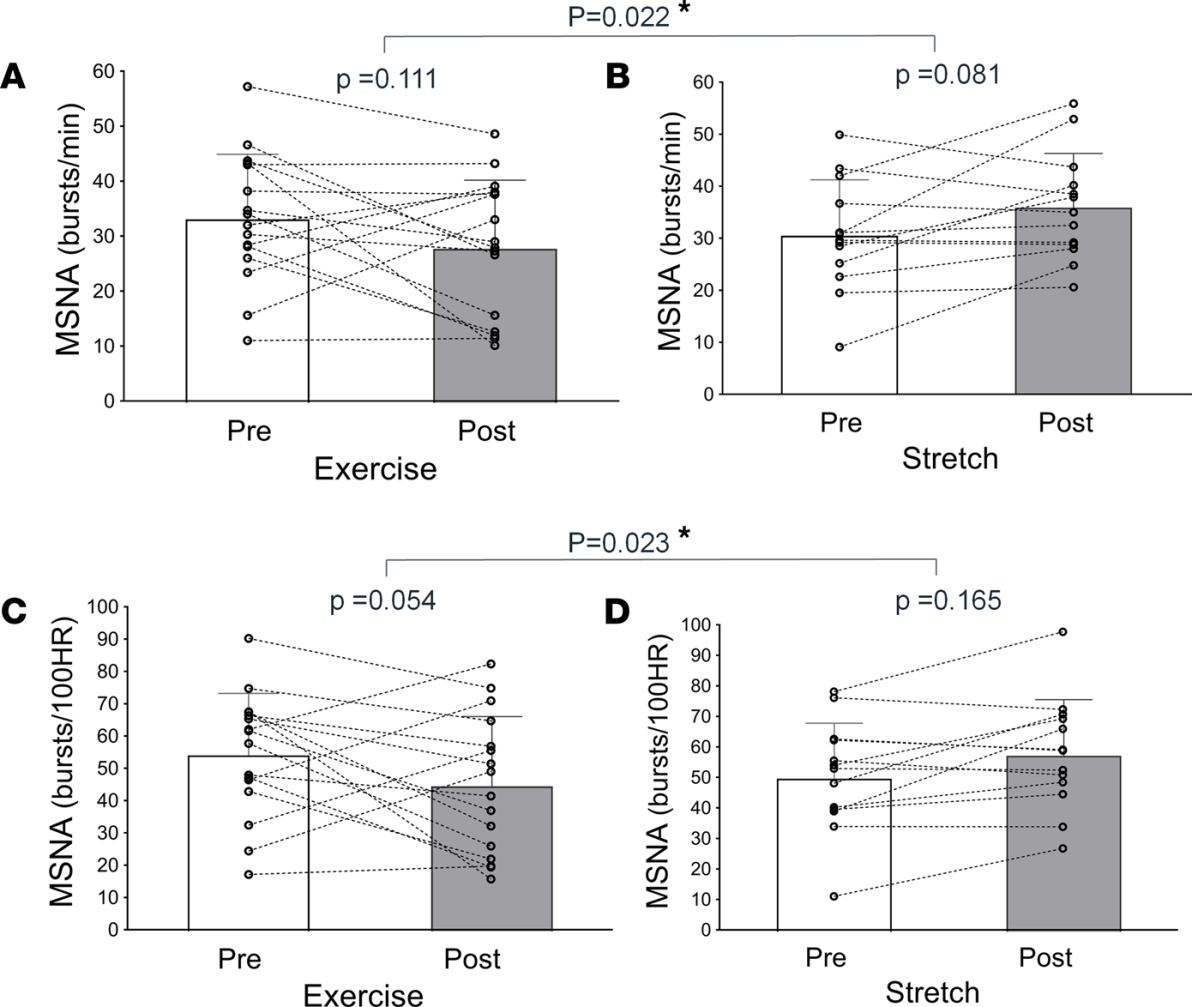
Change in muscle sympathetic nervous activity (MSNA) burst frequency. (**A**–**D**) MSNA burst incidence from baseline (Pre) to 12 weeks (Post) of aerobic exercise training (*n* = 16) versus control stretching (*n* = 13) interventions in patients with chronic kidney disease. Open circles depict individual values for each study participant, and bar graphs depict the mean ± SD values at baseline and 12 weeks within each group. *P* values above bracket denote statistically significant difference in the change from baseline to 12 weeks between groups (group ***×*** time interaction) by 2 way-repeated ANOVA. *P* values on either side of bracket denote within-group comparison from baseline to 12 weeks by independent 2-tailed *t* tests.

The effect of exercise training on MSNA was tested by comparing changes in resting MSNA measured before and after the 12-week intervention between Exercise and Stretching groups in 29 participants (*n* = 16 in Exercise, *n* = 13 in Stretching). There was no difference in MSNA variables between groups at baseline. There was a significant group ***×*** time interaction in MSNA frequency and incidence (*P* = 0.022 and 0.023, respectively; Figure 2) with a decreasing trend in Exercise group (33.5 ± 11.8 [95%CI, 27.6–39.2] to 28.1 ± 12.5 [95%CI, 22.1–34.1] bursts/min, *P* = 0.111; 54.4 ± 19.1 [95%CI, 44.8–64.0] to 44.9 ± 21.5 [95%CI, 34.5–55.3] bursts/100 heart rate [100HR], *P* = 0.054), but an increasing trend in Stretching group (30.6 ± 10.6 [95%CI, 24.1–37.1] to 36.0 ± 10.4 [95%CI, 29.4–42.6] bursts/min, *P* = 0.081; 50.2 ± 18.1 [95%CI, 39.6–60.9] to 57.7 ± 18.3 [95%CI, 46.2–69.2] bursts/100HR, *P* = 0.165) after 12 weeks. Results were similar when adjusted for antihypertensive medication usage or sex.

### Effects of exercise training on arterial stiffness and central and brachial hemodynamics.

The effect of exercise training on vascular function was tested by comparing changes in PWV, AIx, and central and brachial hemodynamics measured before and after the 12-week intervention between Exercise and Stretching groups in 43 participants (*n* = 22 in Exercise, *n* = 21 in Stretching). There were no differences in PWV, AIx, or central and brachial hemodynamics between groups at baseline (Table 2). There was a significant group ***×*** time interaction in markers of aortic wave reflection, including AIx and HR of 75 bpm (AIx@HR75) (*P* = 0.033 and 0.047, respectively), with a significant increase in Stretching group over 12 weeks (*P* = 0.038 and *P* = 0.019, respectively; Table 2); there was no change in Exercise. There were no changes within and between groups over 12 weeks in PWV or brachial and central hemodynamics (Table 2). Results were similar when adjusted for antihypertensive medication usage or sex.

### Association between resting sympathetic nerve activity and arterial stiffness indices at baseline.

**Figure 3:**
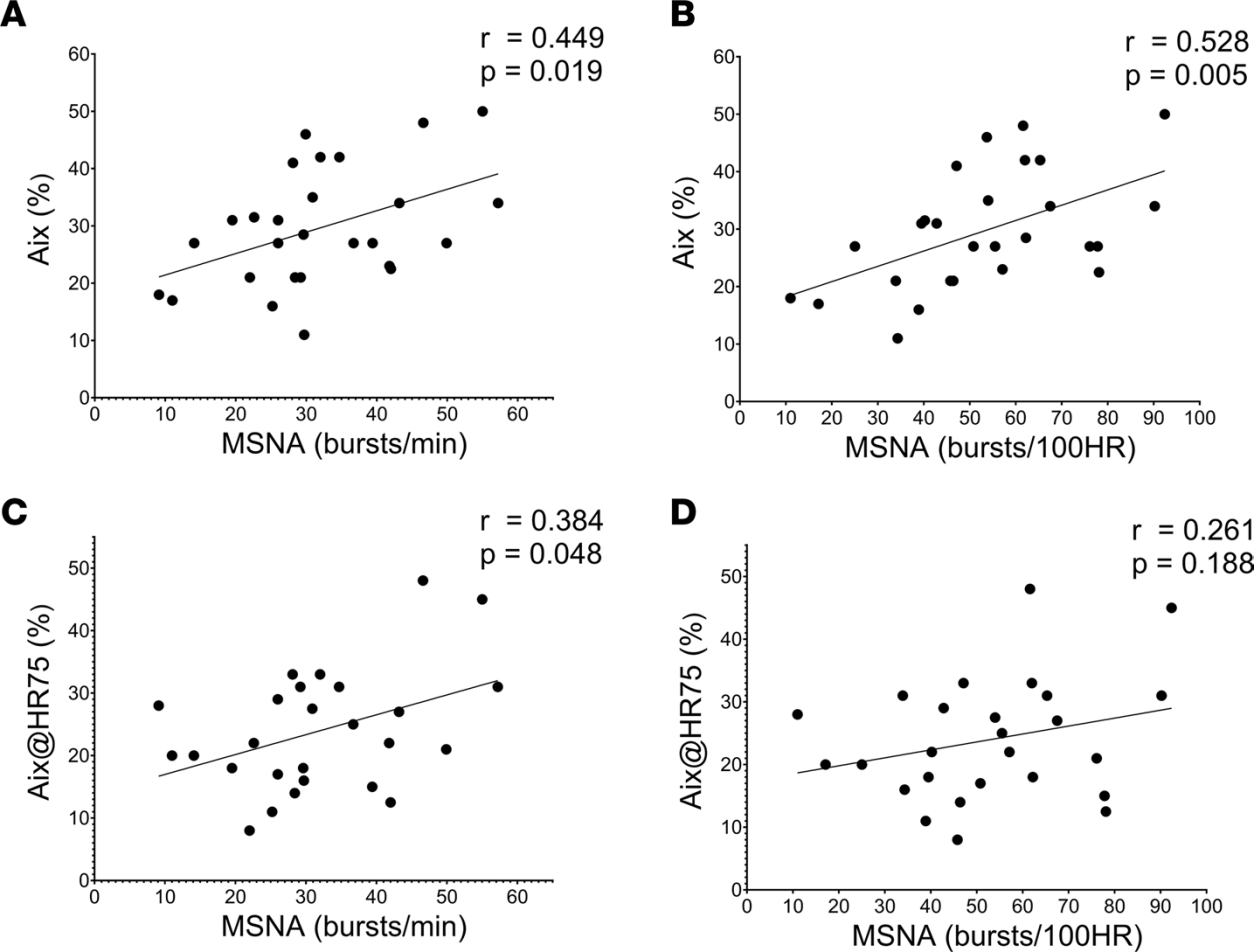
Association between augmentation index (AIx) and muscle sympathetic nervous activity (MSNA). (**A**–**D**)Association between AIx and MSNA burst frequency (**A**) and MSNA burst incidence (**B**) as well as between AIx corrected for heart rate at 75 (AIx@HR75) and MSNA burst frequency (**C**) and MSNA burst incidence (**D**) at baseline in patients with chronic kidney disease (*n* = 27). The *r* and *P* values denote the linear relationship by Pearson correlation tests.

To evaluate the relationship between heightened SNS activity and vascular stiffness, we examined the linear association between MSNA and either PWV or AIx at baseline in the combined CKD groups (*n* = 27). MSNA burst frequency and burst incidence was positively associated with AIx (*P* = 0.019, Figure 3A; *P* = 0.005, Figure 3B) and AIx@HR75 (*P* = 0.048, Figure 3C; *P* = 0.188, Figure 3D). No significant association between MSNA and PWV was found at baseline.

### The impact of baseline MSNA, AIx, and exercise adherence on exercise-induced changes.

**Figure 4:**
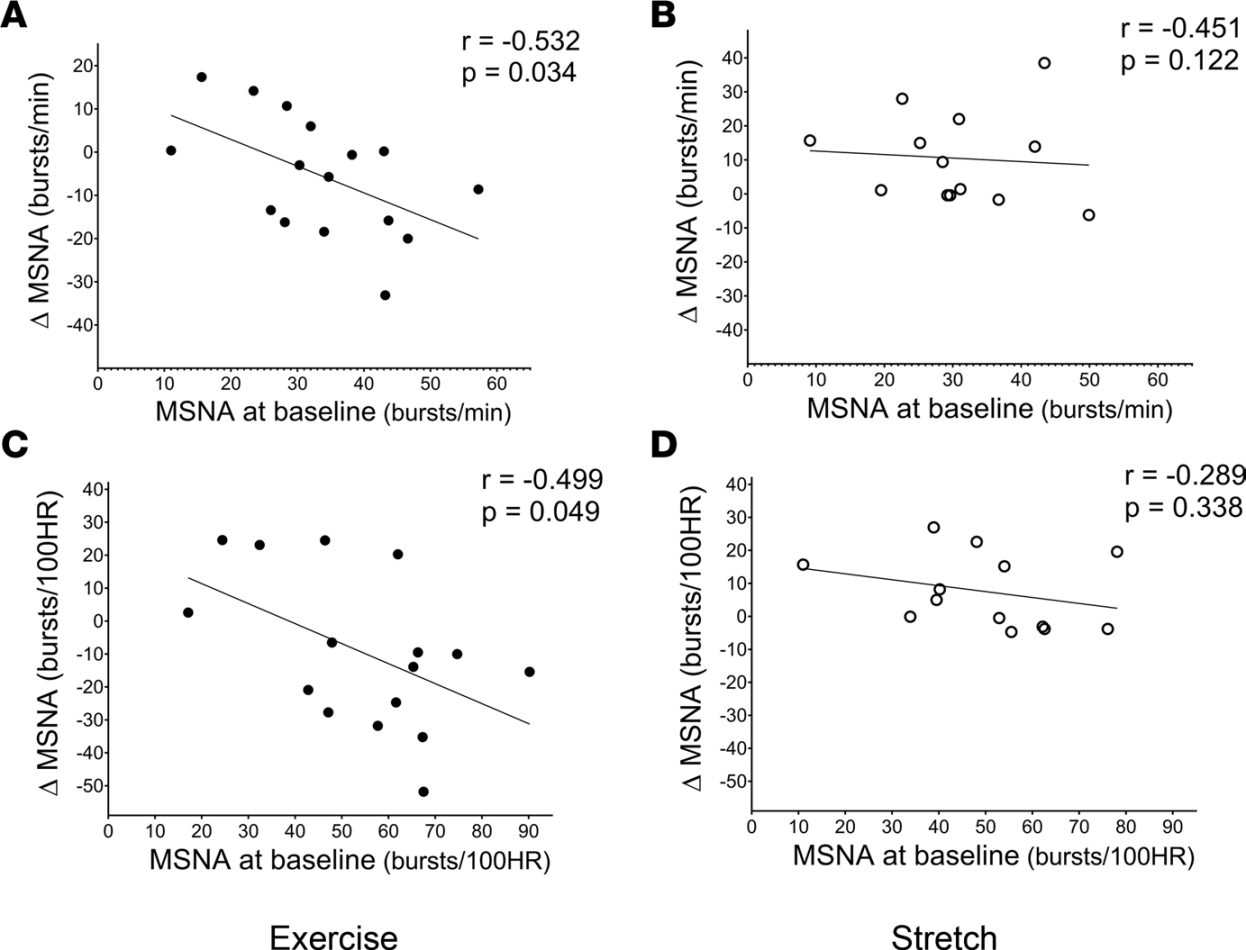
Association between muscle sympathetic nervous activity (MSNA). (**A**–**D**) Association between MSNA burst frequency and MSNA burst incidence at baseline and the change from baseline to 12 weeks in Exercise group (**A** and **C**, respectively; *n* = 16) and in control Stretching group (**B** and **D**, respectively; *n* = 13). The *r* and *P* values denote the linear relationship by Pearson correlation tests. Closed circles depict individual values for each study participant in the Exercise group, while open circles depict individual values for each study participant in the control Stretching group.

**Figure 5:**
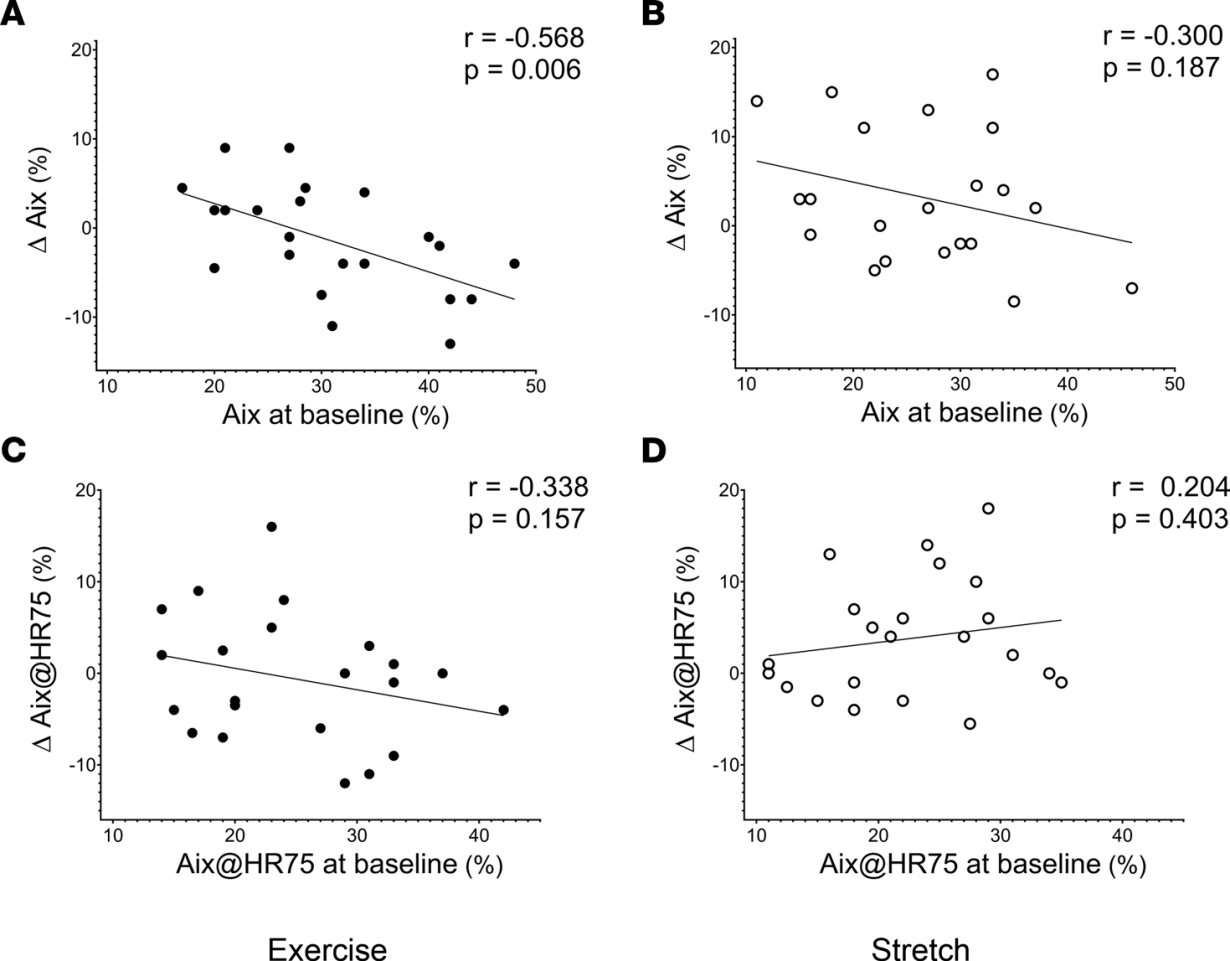
Association between AIx and AIx@HR75. (**A**–**D**) Association between augmentation index (AIx) and AIx corrected for heart rate at 75 (AIx@HR75) at baseline and the change from baseline to 12 weeks in Exercise group (**A** and **C**, respectively; *n* = 22) and in control Stretching group (**B** and **D**, respectively, *n* = 21). The *r* and *P* values denote the linear relationship by Pearson correlation tests. Closed circles depict individual values for each study participant in the exercise group, while open circles depict individual values for each study participant in the control stretching group.

**Figure 6:**
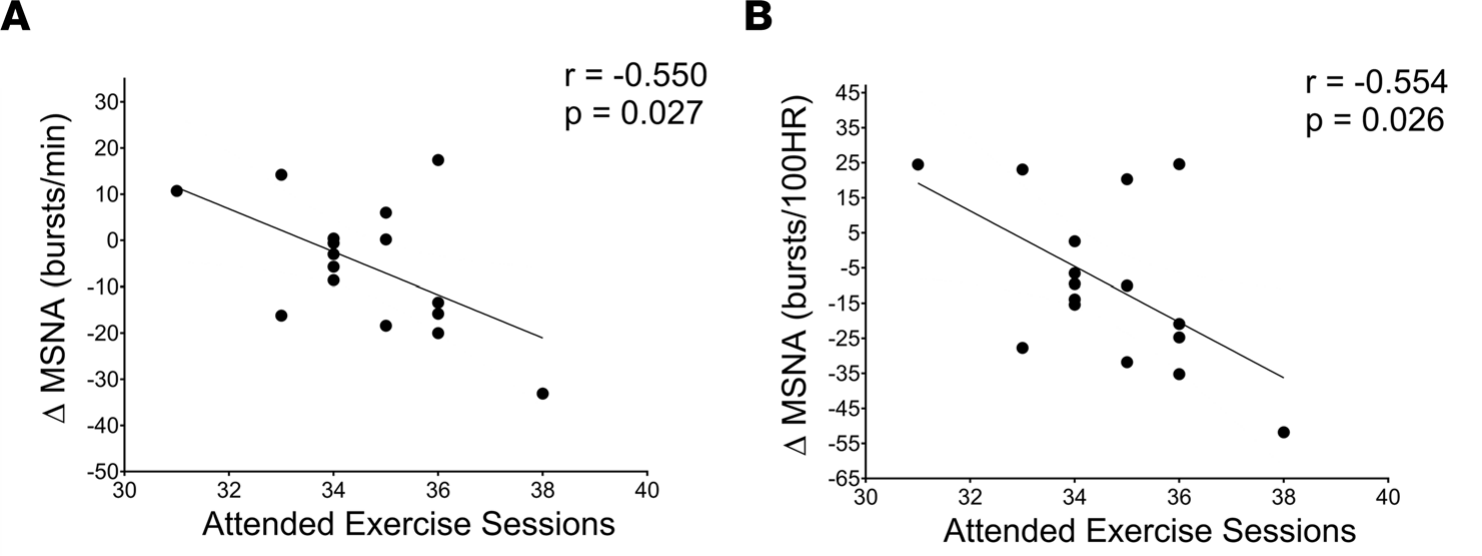
Association between attended exercise sessions and change in muscle sympathetic nervous activity (MSNA). (**A** and **B**) Association between attended exercise sessions and the change from baseline to 12 weeks in MSNA burst frequency and MSNA burst incidence in the Exercise group (**A** and **B**, respectively; *n* = 16). The *r* and *P* values denote the linear relationship by Pearson correlation tests.

We further examined the linear association of baseline levels of MSNA and AIx as well as exercise adherence, with exercise-induced changes in MSNA and AIx within the Exercise group. The magnitude of change in MSNA burst frequency and incidence was inversely associated with baseline MSNA burst frequency (*P* = 0.034; Figure 4A) and incidence (*P* = 0.049; Figure 4C) in the Exercise group, while no significant association was found in the Stretching group (*P* > 0.05; Figure 4, B and D). A similar trend was shown with markers of aortic wave reflection; the magnitude of change in AIx and AIx@HR75 was inversely associated with baseline AIx (*P* = 0.006; Figure 5A) and AIx@HR75 (*P* = 0.157; Figure 5C) in the Exercise group, while no association was observed within the Stretching group (*P* > 0.05; Figure 5, B and D). In addition, the changes in MSNA frequency and incidence were inversely associated with the number of exercise sessions attended (*P* = 0.027, 0.026; Figure 6, A and B, respectively) and the number of exercise sessions after adjustment for VO_2peak_ (*P* = 0.009 and 0.029, respectively) within the Exercise group; however, these associations were not observed within the Stretching group. There was no significant association between the changes in MSNA frequency and the total exercise time (*r* = –0.398, *P* = 0.127) or after adjustment for VO_2peak_ (*P* = 0.140) within the Exercise group.

## Discussion

The present study demonstrates for the first time to our knowledge that exercise training ameliorates worsening of resting SNS overactivity and increased aortic wave reflection associated with vascular stiffness in older sedentary adults with moderate to advanced CKD. This randomized, controlled trial showed that 12 weeks of cycling exercise prevented the progressive increase in resting MSNA and AIx that was observed in the Stretching control group. Furthermore, the magnitude of reduction in MSNA and AIx following exercise training was dependent on baseline levels as well as exercise adherence, with greater reductions observed in participants with higher resting MSNA and AIx, and in those with higher adherence to exercise sessions. Lastly, increased resting MSNA was associated with higher AIx at baseline, suggesting interactive regulation of sympathetic and vascular function in CKD. Our supervised moderate-intensity aerobic exercise intervention was well tolerated with no adverse events and high adherence rates. Although exercise is currently underprescribed in patients with CKD, aerobic exercise training may be a safe and feasible therapeutic intervention to improve autonomic and vascular dysfunction in CKD.

Prior literature has shown that exercise training reduces SNS activity in other clinical populations that are also characterized by SNS overactivity. In patients with heart failure (15–19) and myocardial infarction (31, 32), 3–6 months of exercise training reduced basal MSNA to levels similar to healthy controls. This sympathoinhibitory effect of exercise was independent of sex (18), age (19), beta blocker usage (16), and sleep apnea (17) in cardiac patients as well as in individuals with hypertension (24, 25), metabolic syndrome (20, 21), and obesity (22, 23). We now provide the first evidence to our knowledge for exercise-induced modulation of resting MSNA in CKD, a patient population at increased CV risk by virtue of chronic SNS overactivation. While the control group had an increase in resting MSNA over time, the exercise group demonstrated an amelioration of worsening SNS activation with a trend toward reduction in resting MSNA. Similarly, we observed a worsening of AIx in the control group that was prevented in the exercise group. It may be surprising that the control group exhibited a significant worsening of SNS activity and vascular dysfunction over a 12-week timeframe. However, these data are consistent with prior work supporting the concept that CKD is associated with an accelerated aging phenotype (33–36). In the current study population, older age, comorbidities, and sedentary lifestyle may have contributed to accelerating worsening of autonomic and vascular function in a patient population already at risk for accelerated aging. Indeed, a significant deterioration of vascular endothelial function was observed over 12 weeks of usual care in patients with CKD with similar clinical characteristics to our study population, while no worsening was observed after completion of 12 weeks of supervised aerobic exercise training (30). Our findings suggest that exercise training may mitigate the effects of accelerated aging on neural and vascular function in older, sedentary individuals with CKD, and this may have beneficial effects on long-term CV risk.

We also observed a positive association between MSNA and AIx at baseline in CKD. Although causal mechanisms underlying this relationship remain unexplored, prior work has shown that sympathetic activation influences short-term alterations in arterial distensibility (37–39) and sympathoexcitation induces a marked decrease in arterial compliance (40, 41). In addition, higher MSNA levels were associated with greater reductions in arterial distensibility in heart failure (42, 43) and kidney transplant recipients (44), suggesting that sustained SNS overactivity present in chronic diseases such as CKD may negatively affect arterial elastic properties over time. This association was more pronounced in populations with greater neural control of vasomotor tone, such as patients with heart failure (42, 43), older males, and postmenopausal females (45–47). Our results suggest that CKD may also be characterized by interactive regulation of SNS activity and central wave reflection properties. Given that SNS overactivity (2) and AIx (13, 14) are strong predictors of adverse renal and mortality outcomes in CKD, treatments that target both neural and vascular mechanisms, such as exercise, may have the potential to improve clinical outcomes in CKD.

Interestingly, we observed that the magnitude of reduction in resting MSNA following exercise training was greater in participants with higher resting MSNA at baseline. Previous evidence suggests that the sympathoinhibitory effect of exercise training may be limited to populations with chronically increased SNS activity, while exercise training exerts no effect on sympathetic regulation in healthy participants (48, 49), including middle-aged and older adults (15, 17, 50). Similarly, we observed a greater reduction in AIx following exercise training in those with elevated baseline AIx (51). Our findings implicate that SNS activity and AIx may be biomarkers for identifying patients more likely to derive benefits from exercise training. It is also noteworthy that greater total time of exercise over 12 weeks was associated with a larger reduction in MSNA, highlighting the importance of achieving high adherence rates to produce meaningful health benefits. Indeed, the beneficial effect of exercise training on autonomic function in other at-risk populations appears to be dependent on the exercise intensity and duration (52), although the optimal exercise prescription remains unknown in CKD. Overall, we observed high adherence rates to supervised exercise training with no serious adverse effects, suggesting that exercise training may be a safe, effective, and feasible intervention that can improve autonomic and vascular function in older, sedentary patients with CKD. Our findings may also inform the development and implementation of exercise training protocols in patients with CKD (i.e., renal rehabilitation), as these protocols are poorly developed and sorely lacking when compared with other patient populations (e.g., cardiac rehab and pulmonary rehab). Whether applying different intensities, durations, frequency, and forms of exercise in conjunction with patient risk stratification could generate greater exercise-induced improvements in SNS overactivity and vascular dysfunction in CKD requires further investigation.

Although exercise training prevented the worsening of AIx observed in the control group, we observed no change in PWV with either intervention. While carotid-to-femoral PWV (c-f PWV) is a measurement of large artery stiffness determined mainly by structural remodeling in central elastic arteries, AIx measures the magnitude of augmented pulse wave reflection during the early systolic phase that is driven by both central large artery stiffness as well as accumulated resistance from small to medium peripheral arteries. These smaller arteries are more densely innervated, potentially leading to greater sympathetic control, as compared with large arteries that predominantly rely on structural elastic properties to dampen high pulsatile flow. To support this concept, renal transplant patients with increased MSNA had reduced distensibility of the muscular (medium-sized) artery, but not the elastic type (large-sized) artery (44). Although the precise mechanisms by which exercise training modulates arterial stiffness are unclear, our data suggest that neural mechanisms might be involved, particularly in smaller arteries. Alternatively, it may take a longer training period (greater than 12 weeks) to elicit favorable adaptations in large arteries, as measurable deterioration of PWV is observed to occur over longer timeframes (i.e., 12 months) in CKD (33–35, 53).

### Limitations.

The majority of participants were Black males, and this may limit the generalizability of study results to females and other races. Antihypertensive medications such as angiotensin converting enzyme inhibitors and angiotensin receptor blockers are known to impact sympathetic activity (54); therefore, they may have confounded results. However, there was no difference in medication usage between groups at baseline, and the primary results remained the same when adjusted for medication use. Furthermore, participants maintained a stable medication regimen throughout the study period. Therefore, exercise-induced changes in MSNA and AIx are likely to represent true intervention effects, and the results of this study can be applicable to the general medicated patients with CKD. The present study does not provide information on the potential mechanisms by which exercise modulates SNS activity in CKD. Previous evidence suggests that exercise training may alter levels of angiotensin II, renin, and nitric oxide in brain regions (55–59), regulating SNS control (56, 60) and thereby leading to reductions in resting SNS activity. In addition, improvement in baroreflex sensitivity (24, 31), chemoreflex sensitivity (61, 62), and the exercise pressor reflex (63) could also contribute to exercise-associated neural adaptations and should be investigated in future studies. Although MSNA is the gold-standard assessment of SNS activity in humans, SNS outflow directed to skeletal muscles (i.e., MSNA) may not reflect SNS activation directed to other organs. However, prior studies using simultaneous measurements of circulating norepinephrine (64) and norepinephrine spillover to several internal organs demonstrated good agreement with MSNA (65–67). The linear associations between MSNA and AIx and AIx@HR75 do not establish a causal link. Some medications that are known to alter HR (i.e., beta blockers) may interfere with HR-based target exercise intensity monitoring. However, there were no differences in beta blocker use between groups, and non–HR-based adjustments in exercise intensity (i.e., Borg Rating of Perceived Exertion [RPE] scale that uses the participants’ perceptions of physical exertion on the scales of 0–20) were used when necessary. Finally, we only tested 1 type of exercise paradigm; future studies are needed to determine the ideal exercise prescriptions (i.e., aerobic versus resistance, frequency, and duration) in CKD.

In conclusion, we evaluated the sympathetic- and vascular-modulatory effects of exercise training in CKD, and we demonstrated a protective effect of exercise training on the progression of SNS overactivity and increased aortic wave reflection associated with vascular stiffness. We also observed greater beneficial effects in participants with higher SNS activity and aortic wave reflection at baseline and in those with higher adherence rates; these findings have implications for identifying patients with CKD most likely to benefit from exercise intervention, and they have implications for the need to ensure high adherence rates for maximizing clinical benefits. Given the accelerated aging phenotype in CKD, including worsening SNS overactivation and arterial stiffness in older patients, early initiation and continuous maintenance of an exercise training program may have greater clinical beneficial effects in CKD.

## Methods

### Participants

Sedentary individuals with CKD stages III and IV (estimated glomerular filtration rate [eGFR] between 15 and 59 mL/min/1.73 m^2^), as defined by the CKD-EPI equation (68), were recruited from Emory University clinics and the Atlanta VA Health Care System for participation in this study. All participants were sedentary, defined as engaged in less than 20 minutes twice per week of self-reported physical activity, and had stable renal function (no greater than a decline of eGFR of 1 cc/min/1.73m^2^ per month over the prior 3 months). Exclusion criteria included uncontrolled hypertension (BP > 160/90 mmHg), vascular disease, use of clonidine, clinical evidence of heart failure or active heart disease determined by history, electrocardiogram (ECG) or echocardiogram, ongoing illicit drug use, alcohol use > 2 drinks/day within the past 12 months, diabetic neuropathy, severe anemia (hemoglobin < 10mg/dL), and pregnancy or plans to become pregnant.

### Study design

This randomized, controlled clinical trial was registered at clinicaltrials.gov (NCT02947750). Participants were allocated to an Exercise training group or a control Stretching group using a computer-generated sequence stratified by stage of CKD (Stage IIIA, Stage IIIB, and Stage IV). After obtaining written informed consent, baseline MSNA, brachial BP, body composition, and basic metabolic panel were obtained. On a separate day, baseline aortic hemodynamics, pulse wave analysis, and PWV were measured. All measurements were obtained in a quiet, temperate (21°C) environment, after abstaining from food, caffeine, smoking, and alcohol for at least 12 hours, and after abstaining from exercise for at least 24 hours. Participants were allowed to drink water. After baseline assessments, participants were randomly assigned to 1 of 2 structured, supervised interventions that took place at a frequency of 3 days per week for the duration of 12 weeks (36 sessions total). The aerobic exercise intervention (“spin”) was administered to test the effects of aerobic exercise training on CV health in CKD. The nonaerobic control intervention (stretching and balance) was administered in parallel as the active control intervention conducted within the same group-based environment. At the end of the trial, baseline measurements were repeated under identical conditions within 1 week following the completion of the final training session. Participants were instructed not to change medication regimens, dietary or smoking habits, or home activity levels for the duration of the study. Data from a subset of participants (*n* = 48) were published in a prior publication with a distinctly different experimental question (29).

#### Aerobic exercise intervention.

This intervention consisted of group-based (4–7 participants), progressive, “spin” exercise on a stationary bicycle 3 times per week for 12 weeks (total of 36 sessions) and has been used previously in sedentary older adults (69). The duration of each session began at 20 minutes during week 1 and was progressively increased by 1–2 minutes as tolerated to a goal of 45 minutes per session. The average length of time to reach an exercise duration of 45 minutes was 30 sessions. Each exercise session was supervised and directed by a trained exercise physiologist. Maximal HR was calculated by subtracting the participant’s age from 220, and exercise intensity was individualized to each participant based on maximal HR reserve (HRR) as calculated by the Karvonen method (70); it was targeted to maximize the time that participants maintained HR between 50% and 85% of maximal HRR. The Borg RPE scales between 12 and 15 were also used to guide the targeted intensity. Each session began with a 5-minute warm up, followed by an interval-based, work-out phase that included short bouts of high-intensity sprints and climbs as previously described (69). HR was continuously monitored via a wireless HR monitor (FT7 Polar Heart Rate Monitor) to ensure that all participants maintained their target HR range throughout the duration of each session.

#### Stretching and balance intervention.

This control intervention consisted of balance and stretching exercise for 12 weeks (36 sessions) and matched the active exercise intervention in frequency (3 days/week) and duration (20–45 minutes). Participants performed static and dynamic balance exercises and low-intensity core strengthening exercises using a combination of body weight, light weights, and elastic bands. HR was monitored to gauge exercise intensity and maximize the time that HR was maintained below 50% of HRR. Both exercise and stretching interventions were supervised by an exercise physiologist and were performed at the Movement Studies and Aerobic Exercise Laboratory at the Atlanta VA Health Care System.

### Measurements and procedures

#### BP.

Baseline BP was measured after 5 minutes of rest in a seated position with the arm supported at heart level using an appropriately sized cuff per American College of Cardiology/American Heart Association (ACC/AHA) guidelines (71) using an automated device (Omron, HEM-907XL, Omron Healthcare). Mean arterial BPs (MAP) were calculated as 2/3 diastolic BP (DBP) + 1/3 systolic BP (SBP).

#### MSNA.

Multiunit postganglionic MSNA was recorded directly from the peroneal nerve by microneurography, as previously described (72). Participants were placed in a supine position, and the leg was positioned for microneurography. A tungsten microelectrode (tip diameter 5–15 μm) (Bioengineering, University of Iowa, Iowa City, Iowa, USA) was inserted into the nerve, and a reference microelectrode was inserted s.c. 1–2 cm from the recording electrode. The signals were amplified (total gain of 50,000–100,000 µV/division), filtered (700–2,000 Hz), rectified, and integrated (time constant 0.1 seconds) to obtain a mean voltage display of sympathetic nerve activity (Nerve Traffic Analyzer, model 662C-4; Bioengineering, University of Iowa) that was recorded by the LabChart 7 Program (PowerLab 16sp, ADInstruments). Continuous ECG was recorded simultaneously with the neurogram using a BioAmp system. Beat-to-beat arterial BP was measured concomitantly using a noninvasive monitoring device that detects digital blood flow via finger cuffs and translates blood flow oscillations into continuous pulse pressure waveforms and beat-to-beat values of BP (Finometer, Finapres Medical Systems) (73). Absolute values of BP were internally calibrated using a concomitant upper arm BP reading and were calibrated at the start and every 15 minutes throughout the study. The tungsten microelectrode was manipulated to obtain a satisfactory nerve recording that met previously established criteria (74). After 10 minutes of rest, BP, ECG, and MSNA were recorded continuously for 10 minutes.

#### Arterial waveform assessments.

Central hemodynamics, pulse wave analysis, and PWV were attained in the supine position and estimated noninvasively by an oscillometric cuff-based device (Sphygomocor Xcel, AtCor Medical). To assess central PWV, carotid pulse waves were measured by applanation tonometry, and femoral pulse waves were simultaneously obtained by a partially inflated cuff over the femoral artery at the leg midway between the hip and the knee. The c-f PWV was determined by calculating the ratio of the corrected distance between the pulse-measuring sites to the time delay between the carotid and femoral pulse waves. The distance was measured between (a) the suprasternal notch to the carotid site, (b) femoral artery at the inguinal ligament to the proximal edge of the thigh cuff, and (c) suprasternal notch to the proximal edge of the thigh cuff. Distances a and b were subtracted from distance c and used in the calculation of c-f PWV. Aortic waveform assessments utilized the automatic recording of standard oscillometric brachial BP, followed by partial cuff inflation to subdiastolic pressure levels to capture a brachial artery waveform. The pulse waveform was calibrated to brachial BP, and a validated generalized transfer function was applied to generate the aortic pressure waveform. Pulse wave analysis software was used to compute central BP. Central augmented pressure (AP) was calculated as the difference between the second and the first systolic peaks. AIx, used as an index of aortic wave reflection and a secondary marker of arterial stiffness, was calculated as the ratio of the amplitude of the pressure wave above its systolic shoulder (i.e., the difference between the early and late systolic peaks of the arterial waveform) and the total pulse pressure. AIx values normalized to AIx@HR75 were also reported. All measurements were made in duplicate, and the mean value was used for subsequent analysis.

#### Body composition.

Body composition was estimated using a multifrequency bioimpedance analysis (BIA) with the InBody S10 analyzer (Biospace Co.). All subjects were positioned in a supine position. Contact electrodes were placed on thumbs and middle fingers of both hands and in the space between the malleolus and heel of lower extremities. An alternating intensity of electrical current was applied between the hand and foot of one side. InBody uses an 8-point tetrapolar electrode system method, which assesses the impedance to 6 specific frequencies (1, 5, 50, 250, 500, and 1,000 kHz). The electrical current passes through different tissues depending on the impedance. InBody S10 provides a ratio of extra cellular water/total body water (ECW/TBW) from body segments (both arms, legs, trunks, and whole body) derived from segmental impedance. 

#### Maximal aerobic exercise capacity.

The maximal aerobic exercise test was performed on participants after abstaining from exercise for at least 24 hours before the study. The detailed testing protocol was previously published (29). Participants were instrumented with a 12-lead ECG for assessment of HR and for monitoring by a licensed physician to ensure participant safety during the testing. Expired gases were collected via a metabolic cart (Cosmed Quark CPET) for assessment of peak aerobic capacity (VO_2peak_). After instrumentation, the exercise test commenced in accordance with the modified Balke protocol (75). Briefly, this protocol consisted of 2-minute stages at a fixed speed (3.0 miles/hour) whereas treadmill grade increased progressively with each stage. Testing was terminated when subjects reached volitional fatigue or if a plateau in VO_2_ was achieved so that VO_2_ did not increase further with increasing workloads.

### Data analysis

#### MSNA and ECG data.

MSNA and ECG data were exported from the LabChart data acquisition system to WinCPRS (Absolute Aliens) for blinded analysis. R waves were detected and marked from the continuous ECG recording. MSNA bursts were automatically detected by the program using the following criteria: 3:1 burst/noise ratio within a 0.5-second search window, with an average latency in burst occurrence of 1.2–1.4 seconds from the previous R-wave. After automatic detection, the ECG and MSNA neurograms were visually inspected for accuracy of detection by a single investigator. All MSNA data met previously established quality standards (76, 77). MSNA was expressed as burst frequency (bursts/min) and burst incidence (bursts/100 heartbeats).

### Statistics

We required 36 patients in each group, accounting for a 20% attrition rate, to have 80% power to detect a difference in MSNA change between groups at α = 0.05 based on previous literature (19, 24, 25, 32). The power calculation also accounted for an inability to attain microneurograms in 25% of studies both at baseline and at the end of study. Values are presented as mean ± SD. Differences in participant characteristics between the exercise and the Stretching groups were determined using independent, 2-tailed, *t* tests for continuous variables and χ^2^ tests for categorical variables. The between-group comparison of the change in MSNA and hemodynamic outcomes over 12 weeks were performed using 2 way-repeated ANOVA. Adjustment with potential confounding factors, including the use of antihypertensive medications, was performed. The within-group comparison from baseline to 12 weeks was done using independent, 2-tailed, *t* tests. Pearson correlation test was performed to examine the linear relationship between MSNA and vascular variables, the change in MSNA, the change in AIx and AIx@HR75, and exercise adherence. Multivariable linear regression tests were also used to describe the predictability of the exercise adherence combined with the exercise capacity on MSNA changes in patients with CKD. An α < 0.05 was considered statistically significant for all analyses. All analyses were performed in a blinded manner using SPSS version 26.0 (IBM Corporation).

### Study approval

This study was approved by the Atlanta VA Health Care System Research and Development Committee and the Emory University IRB. Written informed consent was obtained for all study participants, and all study procedures conformed to the standards set forth by the Declaration of Helsinki.

## Author contributions

JJ and JP conceived and designed research; JJ, JDS, DRD, KM, JRN, and JP performed experiments; JJ and JP analyzed data; JJ, JDS, and JP interpreted results of experiments; JJ and JP prepared figures; JJ, JDS, and JP drafted the manuscript; JJ, JDS, DRD, KM, JRN, and JP edited and revised the manuscript; and JJ, JDS, DRD, KM, JRN, and JP approved final version of the manuscript.

## Supplementary Material

ICMJE disclosure forms

## Figures and Tables

**Table 1:**
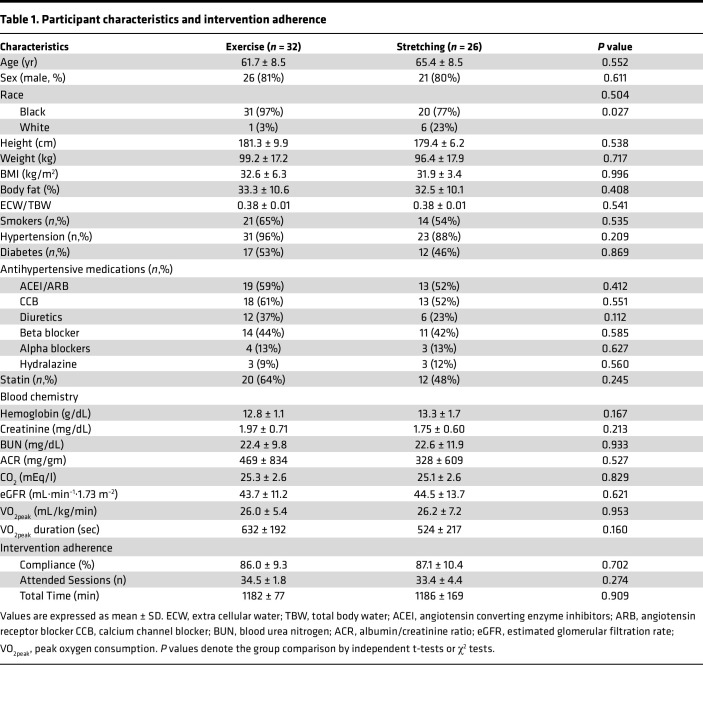
Participant characteristics and intervention adherence

**Table 2:**
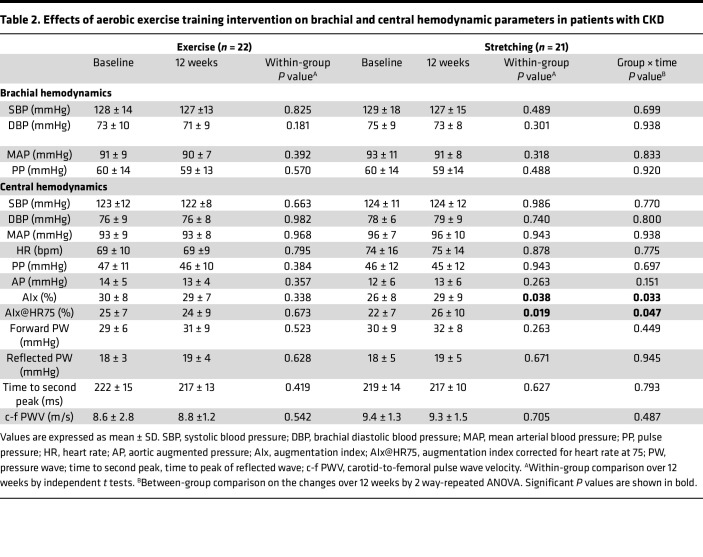
Effects of aerobic exercise training intervention on brachial and central hemodynamic parameters in patients with CKD

## Version 1 02/22/2023

Electronic publication
